# A combined metabolomics and molecular biology approach to reveal hepatic injury and underlying mechanisms after chronic l-lactate exposure in mice

**DOI:** 10.1016/j.csbj.2022.07.034

**Published:** 2022-07-25

**Authors:** Minjian Dong, Qingqing Yi, Danjie Shen, Jiapin Yan, Haowei Jiang, Jiaojiao Xie, Liangcai Zhao, Hongchang Gao

**Affiliations:** a School of Pharmaceutical Sciences, Wenzhou Medical University, Wenzhou 325035, Zhejiang, China; b Oujiang Laboratory (Zhejiang Lab for Regenerative Medicine, Vision and Brain Health), Key Laboratory of Alzheimer's Disease of Zhejiang Province, Wenzhou Medical University, Wenzhou 325035, China

**Keywords:** BDNF, brain-derived neurotrophic factor, MCT, monocarboxylate transporter, GSH, glutathione, ROS, reactive oxygen species, BUN, blood urea nitrogen, SCR, serum creatinine, ALT, alanine aminotransferase, AST, aspartate aminotransferase, H&E, hematoxylin and eosin, PAS, periodic acid-Schiff, NMR, nuclear magnetic resonance, TSP, trimethylsilyl propionate, OPLS-DA, orthogonal projection to latent structures discriminant analysis, VIP, variable importance in projection, MDA, malondialdehyde, DHE, dihydroethidium, VLDL/LDL, very low-density lipoproteins/low-density lipoproteins, AD, Alzheimer's disease, TCA, tricarboxylic acid, Sirt1, silent information regulator 1, CREB, cyclic AMP-responsive element binding protein, Cyp2e1, cytochrome P450 2E1, Nox2, NADPH oxidase-2, Gclc, c-glutamylcysteine ligase catalytic subunit, Sod2, superoxide dismutase-2, LDH, lactate dehydrogenase, Hepatic injury, l-lactate, Nuclear magnetic resonance, Metabolomics

## Abstract

This study aimed to explore whether chronic l-lactate exposure could affect the peripheral tissues of mice and to determine the underlying pathogenesis. Herein, male C57BL/6 mice were divided into control and l-lactate groups. After l-lactate treatment for eight weeks (1 g/kg), metabolic changes in liver, kidney, muscle, and serum samples were determined by ^1^H nuclear magnetic resonance (^1^H NMR)-based metabolomics. Additionally, organ function was evaluated by serum biochemical and histopathological examinations. Reactive oxygen species (ROS) levels were measured using dihydroethidium staining; levels of signals involved in lactate metabolism and ROS-related pathways were detected using western blotting or polymerase chain reaction. Apoptosis was detected by TUNEL-fluorescence staining. Metabolomic analysis revealed that l-lactate mice showed decreased levels of glutathione (GSH), taurine, ATP, and increased glucose content, compared to control mice. Furthermore, l-lactate mice presented significantly higher serum levels of alanine aminotransferase and aspartate aminotransferase and increased glycogen content in hepatic tissues, compared to control mice. l-lactate mice also had a greater number of apoptotic nuclei in the livers than controls. Moreover, l-lactate exposure reduced mRNA and protein levels of superoxide dismutase-2 and c-glutamylcysteine ligase, elevated levels of cytochrome P450 2E1 and NADPH oxidase-2, and increased the protein expressions of LDHB, Bax/Bcl-2, cleaved caspase-3, and sirtuin-1 in hepatic tissues. Together, these results indicate that chronic l-lactate exposure increases oxidative stress and apoptosis in hepatocytes via upregulation of Bax/Bcl-2 expression and the consequent mitochondrial cytochrome-C release and caspase-3 activation, which contributes to the pathogenesis of hepatic dysfunction.

## Graphical abstract

**Figure:**
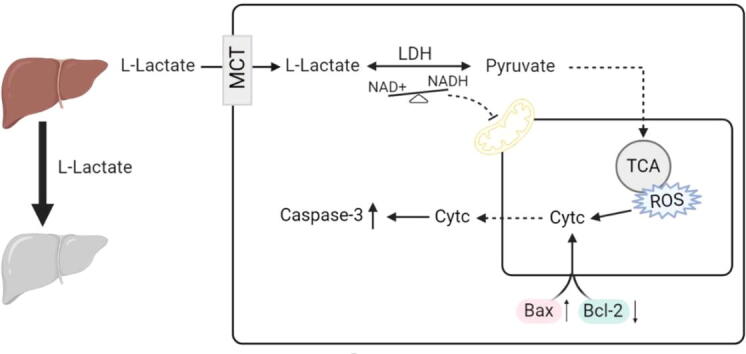

## 1 Introduction

Hyperlactatemia occurs when lactate production exceeds lactate removal. The body produces l-lactate, which can be absorbed and utilized as an energy source, under physiological or pathological conditions such as physical exercise, hypoxia, diabetes, and cancer [1], [2], [3], [4]. l-lactate can be transported to various organs, such as the skeletal muscle, liver, kidney, and heart, where it effects different responses [5], [6]. Furthermore, current research has suggested that l-lactate may function as a signaling molecule [7], [8].

l-lactate has been shown to precisely control gene and protein expression [5], [6], [9]. In the brain, systemic delivery of l-lactate through intraperitoneal injections induces hippocampal expression of brain-derived neurotrophic factor (BDNF) and improves learning and memory creation [10]. In mice, peripheral administration of l-lactate also produces antidepressant-like effects, especially in terms of serotonin receptor trafficking, astrocyte functions, neurogenesis, nitric oxide synthesis, and cAMP signaling [11]. In skeletal muscle, l-lactate controls the expression of several proteins involved in mitochondrial biogenesis [12], while in adipose tissue, extracellular l-lactate mediates antilipolytic action by activating the Gi-coupled receptor GPR81 via downregulation of cAMP levels [13] or p38-MAPK-dependent FGF21 expression [14]. Furthermore, l-lactate can enter cells through monocarboxylate transporters (MCTs), thereby inducing ATP production [8], [15] and changes in cellular redox conditions by altering the NAD^+^/NADH ratio [10], [16]. Recently, San-Millan and his colleagues observed chronic l-lactate exposure decreases mitochondrial function of cardiomyocytes by inhibition of fatty acid uptake [17]. However, further systematic research needs to be performed to determine the impact of l-lactate on peripheral organs and the molecular mechanisms underlying its effects.

Metabolomics is a systems biology-based method that is used to determine a large number of endogenous metabolites in order to clarify the intrinsic differences in samples and to identify significant variables as characteristic biomarkers [18], [19], [20], [21], [22]. Additionally, nuclear magnetic resonance (NMR) analysis has high reproducibility and is quantitatively advantageous, as it requires minimal sample pretreatment [23]. Therefore, using NMR-based metabolomics and molecular-biology techniques, we previously screened several metabolites that were closely related to the pathogenesis of diabetic complications [24], [25], [26], [27]. These studies demonstrated that the combination of systems biology-based and molecular biology-based approaches could be used to identify precursor–product relationships that drive perturbations, thereby providing a better understanding of the pathogenic mechanisms of diseases.

In the present study, we established a chronic l-lactate-exposure mouse model. Thereafter, we integrated the aforementioned metabolomics and molecular biology technologies to explore whether exogenous l-lactate administration had an effect on liver, kidney, muscle, and serum samples and to determine the mechanism underlying these observed effects.

## 2 Materials and methods

### 2.1 Animal treatment

Eight-week-old male C57BL/6 mice (18–22 g) were purchased from Shanghai Slack Laboratory Animal Co., ltd. and were raised at the SPF Laboratory of the Experimental Animal Center of Wenzhou Medical University. All mouse experiments were performed according to the National Institutes of Health Laboratory Animal Care and Use Guidelines and were approved by the Animal Health and Use Committee of Wenzhou Medical University (document number: wydw 2018–029). The feeding environment guaranteed standard temperature and humidity from 8:00 in the morning to ensure a 12-h/12-h light/dark cycle with a provision for standard animal feed and drinking water. We also took steps to minimize the number of mice used and the suffering of the animals in the study.

### 2.2 Animal experiments and sample collection

After adaptive feeding for 1 week, the mice were randomly divided into a control group and an l-lactate group. Each mouse was administered sterile saline or l-lactate for eight consecutive weeks (1 g/kg, i.p.), as previously described [9]. Blood glucose obtained from tail and body weight values were measured weekly, respectively. Then, the mice were euthanized and their the kidney, liver, and muscle (gastrocnemius) tissues were harvested 0.5 h after the administration, immediately snap-frozen in liquid nitrogen, and stored at –80 °C. The blood samples collected from the abdominal aorta were centrifuged at 3000 × *g* for 15 min at 4 °C to obtain serum samples that were subsequently used for biochemical analyses and ^1^H NMR spectroscopy. For histopathological assessments, the tissues were isolated and fixed in 4 % paraformaldehyde for at least 24 h. Thereafter, these samples were embedded in paraffin and sectioned (thickness, 6 μm) using a slicing microtome (Leica, Germany).

### 2.3 Serum biochemical analysis

Renal function was evaluated by measuring blood urea nitrogen (BUN) and serum creatinine (SCR) levels. Hepatic function was evaluated by measuring alanine aminotransferase (ALT) and aspartate aminotransferase (AST) levels, which were detected using a commercial biochemical kit (Jiancheng Bioengineering Institute, Nanjing, China).

### 2.4 Histopathological examination

The 6-μm thick sections of paraffin-embedded formalin-fixed kidney tissues were stained with hematoxylin and eosin (H&E) and periodic acid-Schiff (PAS) stain. The PAS stain is routinely used for visualizing sugar moieties and comparable to H&E staining, provides insight into the general tissue structure, combined with specific information on granular glycogen deposits. The number of glycogen granules stained in the sections was scored under light microscopy by blinded observation. The degree of glycogen deposits was scored using three randomly selected fields for each section, as follows: score 0, colorless cytoplasm and no visible glycogen granules; score 1, reddish cytoplasm or with a small number of red granules, usually<10; score 2, red cytoplasm or more than 10 red glycogen granules; score 3, dark red cytoplasm or granules aggregated into red blocks. The images were magnified at 200×, and six fields were randomly captured.

### 2.5 Acquisition of ^1^H NMR spectra

Samples were prepared and ^1^H NMR spectra were acquired as reported previously [28], [29], [30]. Briefly, before NMR analysis, 200 μL of thawed serum samples were mixed with 400 μL of phosphate buffer (0.2 mM Na_2_HPO_4_/NaH_2_PO_4_, pH 7.4) and 60 μL of deuterated water (D_2_O) to operate deuterium locking and to define more accurate chemical shifts. The frozen kidney, liver, and muscle tissues were weighed and placed in a tube. Ice-cold methanol (4 mL/g) and distilled water (0.85 mL/g) were added to the tissue samples, homogenized at 4 °C after thawing, and mixed. Chloroform (2 mL/g) and distilled water (2 mL/g) were added to the homogenate and mixed again. The homogenate was centrifuged at 1000 × *g* for 15 min at 4 °C. The supernatant was extracted and lyophilized for 24 h.

The tissue extracts were resuspended in 500 μL of D_2_O containing sodium trimethylsilyl propionate-d4 (TSP, 0.42 mM), and the supernatant was transferred to 5-mm NMR tubes. ^1^H NMR spectra were acquired at 25 °C on a Bruker AVANCE III 600 MHz NMR spectrometer equipped with a triple resonance probe. A one-dimensional ZGPR pulse sequence was used to suppress water in the extracts. For each sample, 128 transients were collected into 64 k data points with a spectral width of 12,000 Hz and a relaxation delay of 6 s.

The spectra were phased and baseline corrected; thereafter, the datapoints were reduced to 11,000 integrated regions of 0.001-ppm width corresponding to the region of *δ* 10 to −1 for the multivariate pattern recognition analysis. For NMR spectra recorded in the extracts, the region of approximately *δ* 4.69–5.04 was removed to eliminate residual water resonance. The remaining segments were normalized to the total sum of the spectral intensities to compensate for variations in the total extract volume. The normalized integral values were then subjected to multivariate pattern recognition analysis using SIMCA-P^+^ V12.0 (Umetrics, Umea, Sweden). Following a preliminary principal components analysis, supervised orthogonal projection to latent structures discriminant analysis (OPLS-DA) was also performed for class discrimination and biomarker identification [31].

### 2.6 Multivariate pattern recognition analysis

Data were visualized with a principal component (PC) score plot of the first two principal components (PC1 and PC2) to provide a 2-D representation of the information. Each point represents the individual spectrum of the sample. OPLS-DA revealed differences between the groups, which were necessary to eliminate outliers and enhance the quality of the model. The loading plots, which were assessed by the value of the correlation coefficient, |r|, can be used to identify the characteristic variables that contribute to the separation of metabolic profiles. The scores and loading plots complemented each other. Meanwhile, the following parameters were calculated: R^2^X and R^2^Y, which explained the variance in the matrix of NMR data and class membership, respectively; and Q^2^, which quantifies the model's predictive capability, commonly used to indicate the quality of the model [32]. Values of R^2^Y and Q^2^ that are close to 1.0 represent an excellent model. Furthermore, the significance of the models was tested by CV-ANOVA at the level of *p* < 0.05, using SIMCA software [33]. Other variable filter parameters used included variable importance in projection (VIP) scores and loading values.

### 2.7 Measurement of malondialdehyde (MDA), reduced glutathione (GSH), and oxidized glutathione disulfide (GSSG) levels

Hepatic MDA,GSH and GSSG levels were measured according to a previous study [34]. Briefly, the liver tissues were minced in 300 μL PBS buffer (pH 7.4) and centrifuged at 1000 × *g* for 15 min at 4 °C. MDA reacts with thiobarbituric acid (TBA) at 90–100 °C under acidic conditions. This reaction yielded a pink MDA-TBA conjugate, which was measured using a 532-nm microplate reader (Spectra Max M5). GSH was reacted with dithiobis nitrobenzoic acid (DTNB) at room temperature for 5 min. This reaction yielded a yellow substance, which was measured at 405 nm. The data were classified according to the protein concentration of each sample. Protein concentration was measured using a Bradford protein assay kit (P0006; Beyotime Institute of Biotechnology, Beijing) according to the manufacturer’s instructions.

### 2.8 NAD^+^ and NADH contents determination

The hippocampal tissues samples (10–20 mg) were homogenized, and the NAD^+^/NADH Assay Kit with WST-8 (Beyotime Institute of Biotechnology, Shanghai) was used to detect the NAD^+^ and NADH contents.

### 2.9 Histology assay

Reactive oxygen species (ROS) levels were measured using a dihydroethidium (DHE) assay kit (S0063, Beyotime Institute of Biotechnology, Shanghai, China) according to the manufacturer's instructions. Briefly, the liver sections were immersed in 10 μM DHE in a humidified environment at 37 °C for 30 min. Dihydroethidium is oxidized by superoxide to form ethidium, which binds to DNA in the nucleus and emits red fluorescence. Terminal deoxynucleotidyl transferase dUTP nick end labeling (TUNEL) staining in liver sections was performed using a Cell Death Detection Kit (C1088, Beyotime Institute of Biotechnology). The sections were coverslipped and visualized under a fluorescence microscope (Nikon, Japan). Digital images were processed using Image J software (1.48v, National Institutes of Health, USA). The threshold of fluorescence intensity was automatically defined by Image J software, which should calibrate all optical densities to the same standard.

### 2.10 Real-time polymerase chain reaction

Total RNA was isolated using TRIzol reagent (Invitrogen, USA) and converted to complementary DNA using the PrimeScript RT reagent kit (RR037A; Takara Biomedical Technology, Beijing, China). PCR was performed using TB Green Premix Ex Taq mix (RR820A, Takara Biomedical Technology) and a CFX96 PCR thermocycler (Bio-Rad, USA). Mouse primer sequences for the target genes are presented in Supplementary Table 1. Gene expression was normalized to the expression of *β-actin*.

### 2.11 Extraction of mitochondrial protein

Tissues were lysed in RIPA buffer (P0013E-2, Beyotime Institute of Biotechnology). The homogenates were incubated on ice for 15 min, and lysates were centrifuged at 12,000×*g* for 20 min; the supernatant was used as the total protein. The mitochondrial protein was extracted using a mitochondrial isolation kit (C3606, Beyotime Institute of Biotechnology). Briefly, 100 mg of liver tissue was mixed with 250 μL reagent A and was ground. This mixture was carefully transferred and centrifuged at 3500×*g* for 10 min. The supernatant was collected and centrifuged at 12,000×*g* for 10 min. The supernatant was the cytoplasm, while the precipitate was the mitochondrial part.

### 2.12 Western blotting

Total cellular protein, cytoplasm, or mitochondrial protein extracted from liver tissues were resolved by SDS-PAGE and transferred to PVDF membranes, which were subsequently probed with primary antibodies, including those for caspase3, cleaved caspase3, lactate dehydrogenase-B (LDH-B), B cell lymphoma/leukemia 2 (Bcl2), Bcl2-associated × (Bax), cytochrome *c* (Cytc), cytochrome *c* oxidase (Cox) IV, silent information regulator 1 (Sirt1), cyclic AMP-responsive element binding protein (Creb), p-Creb (Cell Signaling Technology, USA), GAPDH (Abcam, USA), and β-actin (Abcam, USA). The protein bands were visualized with EZ EZ-ECL enhanced chemiluminescence reagents (Biological Industries, Isreal) and quantified using Image J software.

### 2.13 Statistical analysis

Data are presented as the mean ± standard deviation (S.D.) unless otherwise specified. All data were analyzed using one-way analysis of variance (ANOVA) followed by Bonferroni’s *post-hoc* test. Statistical significance was set at *p* < 0.05. SPSS for Windows (Version 13.0, SPSS Inc., Chicago, IL, USA) was used for the statistical tests. The figures were generated using Prism software (GraphPad Software, San Diego, USA).

## 3 Results

### 3.1 Altered l-lactate levels in the serum, kidney, liver, and muscle tissues of mice after chronic l-lactate exposure

**Fig. 1:**
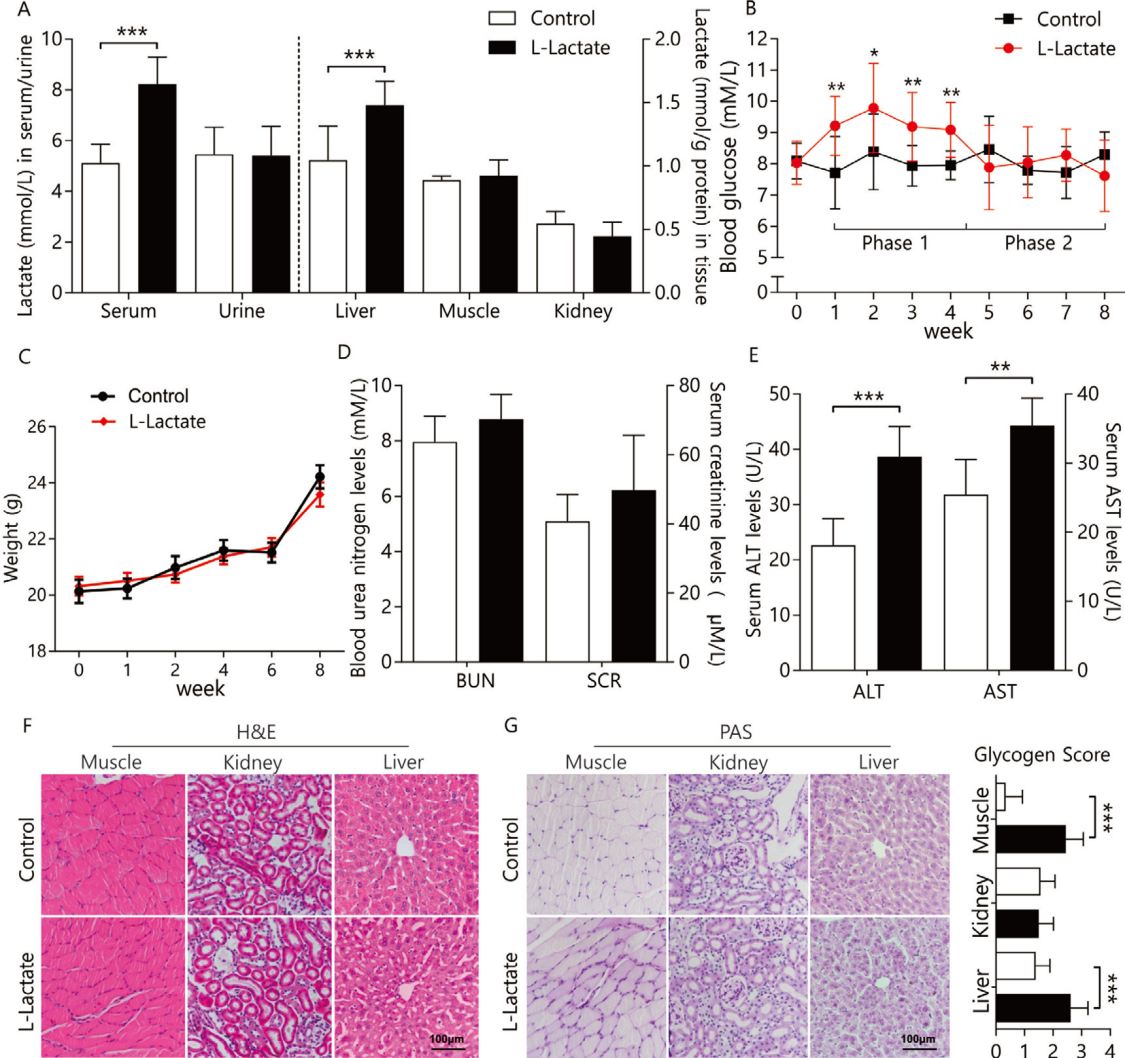
Chronic l-lactate administration for eight weeks causes specific hepatic damage in mice. (A) l-lactate levels in serum, muscle, liver, urine, and kidneys from l-lactate and control mice (n = 6). (B) Blood glucose levels per week (n = 6). (C) Body weights per week (n = 10). (D-E) Blood urea nitrogen (BUN), serum creatinine (SCR), alanine aminotransferase (ALT), and aspartate aminotransferase (AST) levels (n = 6). (F) Representative hematoxylin and eosin (H&E) staining (n = 3). (G) Representative periodic acid-Schiff (PAS) staining and glycogen score on a scale of 0–3 (n = 3). *P < 0.05, ** *P* < 0.01, and *** *P* < 0.001.

After chronic l-lactate administration for eight weeks, we found that blood l-lactate levels significantly increased compared with those in age-matched normal mice, while urine l-lactate levels showed no changes. It was reported that blood lactate concentration reached 18 mM at 5 min after the injection and returned to the baseline level after 3 h [9]. At the same time, we also found significantly elevated l-lactate levels in mouse livers but not in the kidney and muscle tissues (Fig. 1A), suggesting that l-lactate accumulated mainly in the liver tissues after chronic l-lactate exposure.

### 3.2 Effects of chronic l-lactate exposure on blood glucose, and body weight in the mice

In the liver, l-lactate can be converted to glucose through the gluconeogenesis pathway; therefore, we determined blood glucose levels in the mice. Interestingly, there were two stages after l-lactate exposure (Fig. 1B): the stage with significantly elevated glucose levels, which occurred during weeks 1–4 (phase 1), and the stage with no changes in glucose levels, which occurred during weeks 5–8 (phase 2). In addition, the body weight of the l-lactate mice showed no changes during the entire period compared to the body weight of the control mice (Fig. 1C).

### 3.3 Examination of hepatic injuries after chronic l-lactate exposure

To investigate the effects of chronic l-lactate exposure on organ function, serum biochemistry and histopathology were performed. The data showed that ALT and AST levels (clinical indicators of hepatotoxicity [35]) were elevated in the l-lactate mice compared to the controls, while BUN and SCR levels did not change significantly, indicating possible liver injuries under chronic l-lactate exposure (Fig. 1D-E). Furthermore, H&E staining showed infiltration of numerous inflammatory cells into liver lobules, loss of sinusoidal cells, and tissue destruction in the liver, while PAS staining showed glycogen accumulation in the liver of the l-lactate mice. Furthermore, we did not observe any morphological changes in H&E and PAS staining in the kidneys, which suggested no histopathological changes occurred in the kidney after l-lactate exposure. Taken together, chronic l-lactate exposure leads to hepatic pathological injuries and dysfunction, but does not affect other peripheral organs or tissues.

### 3.4 ^1^H NMR-based analysis of l-lactate exposure on the metabolome of mice

**Fig. 2:**
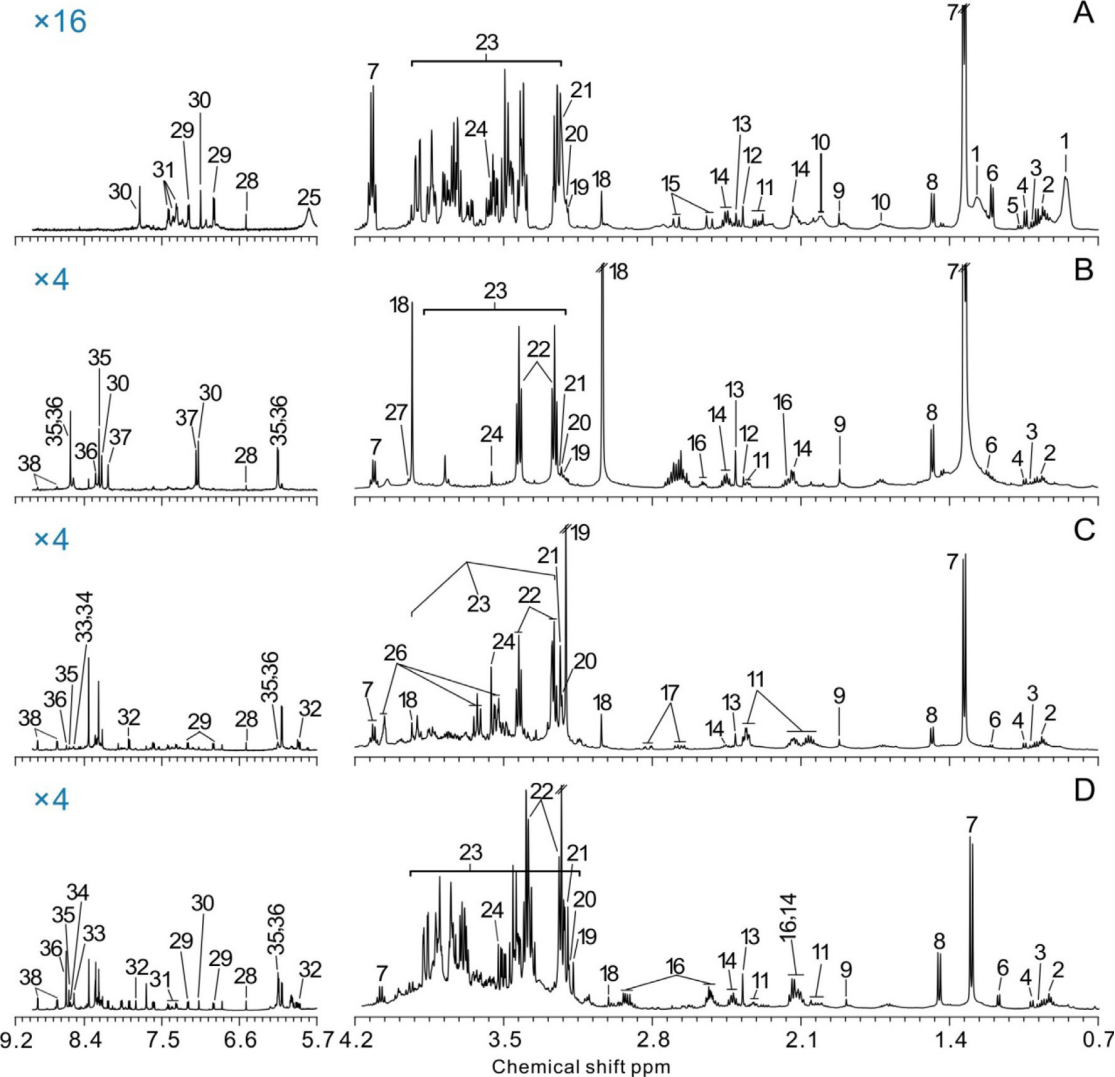
Typical ^1^H NMR spectra of serum (A), muscle (B), kidney (C), and liver (D) samples from one control mouse. Key: 1, VLDL/LDL (Very-low-density lipoprotein/low-density lipoprotein); 2, leucine; 3, isoleucine; 4, valine; 5, isobutyrate; 6, 3-hydroxybutyrate; 7, lactate; 8, alanine; 9, acetate; 10, NAC; 11, glutamate; 12, pyruvate; 13, succinate; 14, glutamine; 15, citrate; 16, GSH; 17, aspartate; 18, creatine; 19, choline; 20, phosphocholine; 21, *sn*-glycerol-3-phosphocholine; 22, taurine; 23, glucose; 24, glycine; 25, urea; 26, myo-inositol; 27, phosphocreatine; 28, fumarate; 29, tyrosine; 30, histidine; 31, phenylalanine; 32, uridine; 33, ADP (adenosine diphosphate); 34, ATP (adenosine triphosphate); 35, IMP (inosinic acid); 36, AMP (adenosine monophosphate); 37, histamine; 38, niacinamide.

**Fig. 3:**
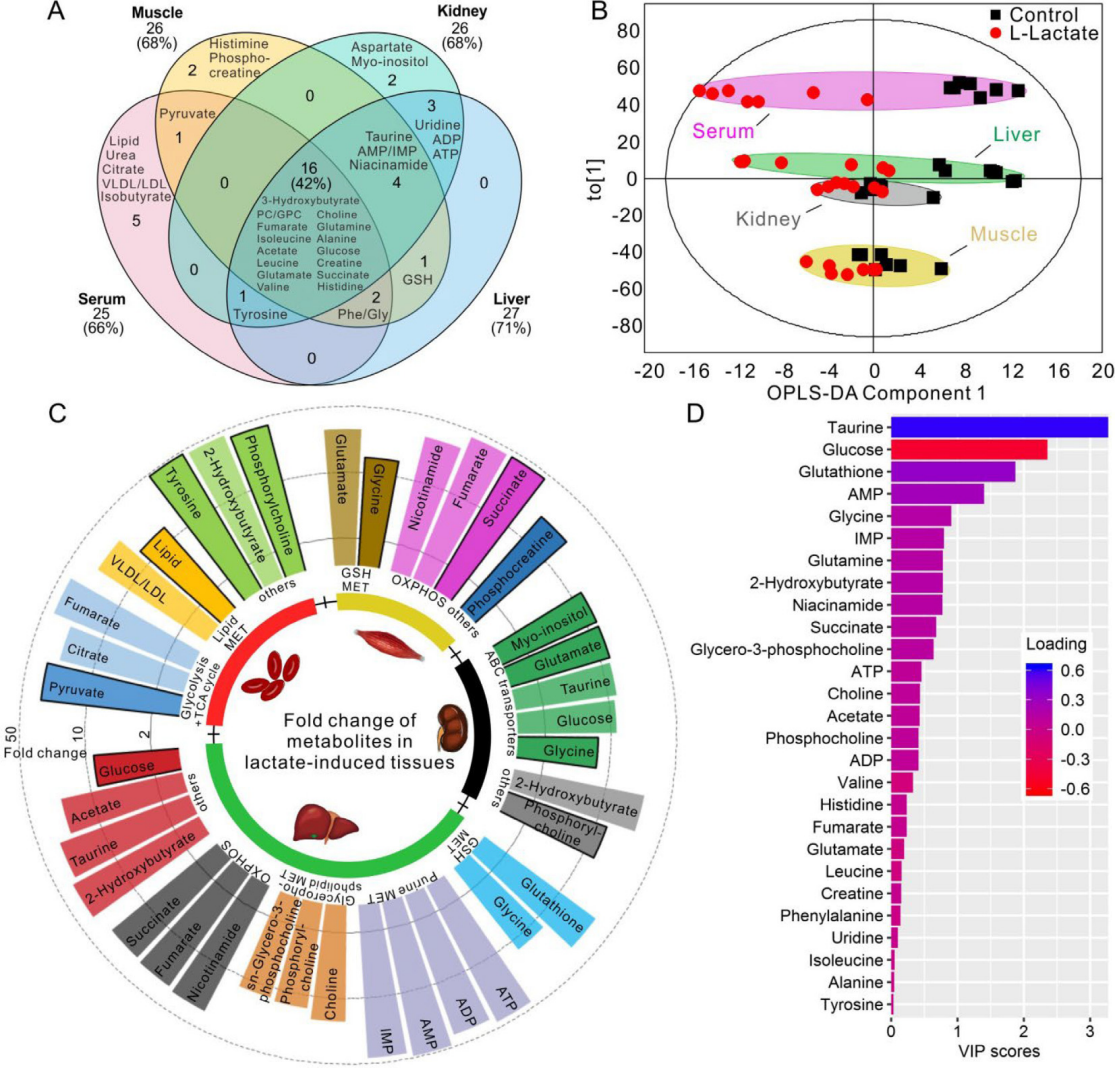
Differential metabolic profiles and characteristic metabolites in tissues from control and l-lactate mice (n = 7). (A) Venn diagram displaying metabolites in different samples. (B) Score plot of the OPLS-DA model shows distinct clustering of metabolites for different groups of mice. Each point represents one variable (metabolite). Metabolic patterns were also grouped by different colored circles according to the tissues. (C) Fold change of metabolites in l-lactate mice, compared to controls, and the corresponding metabolic pathway. The darker border represents a positive fold change compared to controls, while no border indicates a negative fold change compared to controls. (D) VIP rank-score of metabolites of hepatic extracts. Keys: MET, metabolic pathway; OXPHOS, oxidative phosphorylation.

To determine the metabolomic changes involved in l-lactate exposure and to identify potential metabolic biomarkers, we analyzed the ^1^H NMR spectra obtained from the serum, muscle, kidney, and liver extracts of the mice (Fig. 2). We identified 38 metabolites in the spectra, including very low-density lipoproteins/low-density lipoproteins (VLDL/LDL), amino acids (leucine, isoleucine, valine, alanine, glutamate, aspartate, taurine, glycine, tyrosine, histidine, phenylalanine), glucose, tricarboxylic acid (TCA) intermediates (pyruvate, succinate, citrate, and fumarate), organic acids (l-lactate, isobutyrate, 2-hydroxybutyrate, acetate, creatine, phosphocreatine), lipids (lipid, choline, phosphocholine, *sn*-glycerol-3-phosphocholine), ribonucleotides (ADP, ATP, IMP, AMP), amides, and amines (glutamine, histamine, glutathione [GSH], niacinamide), and others (myo-inositol, uridine, urea). The metabolites were classified using Venn diagrams, in which 16 metabolites (42 %) were detected in all tissues (Fig. 3A). We only detected urea, citrate, VLDL/LDL, and isobutyrate in serum samples, while GSH was only found in liver and muscle extracts.

Firstly, PCA was performed for pairwise comparisons of all samples, which showed the clear separation of groups in serum and liver extracts, but not in kidney and muscle extracts (Supplementary Fig. 2). Furthermore, OPLS-DA demonstrated clear differences in the serum and liver extracts, consistent with the PCA data (Fig. 3B). The quality of the PCA and OPLS-DA models generated was indicated by the *R^2^, Q^2^*, and *P* values (Supplementary Table 2). In the PCA model, the *R^2^*X value of the kidney model was<0.50, while in the OPLS-DA models, *R^2^*X of the kidney and muscle was<0.50, indicating the poor robustness and reliability of these models. The cross-validation *P* values of the muscle and kidney models were more than 0.05, further confirming the overfitting of the mathematical models (Supplementary Table 2). Conversely, the parameters that evaluated the PCA and OPLS-DA models in the serum and liver samples revealed that these models were robust and credible. All the results indicated that chronic l-lactate exposure can lead to metabolic changes in the liver and serum tissues, but not in the kidney and muscles.

### 3.5 Identification of characteristic metabolites involved in chronic l-lactate exposure

The characteristic metabolites are illustrated in the color-coded coefficient plots of PLS-DA with their coefficients |r| greater than 0.56, which were also statistically significant (Supplementary Fig. 3, Supplementary table 3). We observed that serum citrate, fumarate, VLDL/LDL, and 2-hydroxybutyrate levels were decreased and pyruvate, tyrosine, and phosphorylcholine levels were increased in the l-lactate mice than in the controls (Fig. 3C, Supplementary Fig. 3). In the liver of l-lactate mice, we only observed increased glucose levels, while the levels of most of the detected metabolites were decreased significantly, including 2-hydroxybutyrate, succinate, GSH, choline, phosphocholine, GPC, taurine, glycine, fumarate, ADP, AMP, ATP, IMP, and niacinamide (Fig. 3C, Supplementary table 3).

According to data from the liver OPLS-DA model, we determined and ranked the VIP scores of the metabolites; taurine, glucose, and glutathione had the highest values (Fig. 3D). Further analysis indicated that serum glucose levels were positively correlated, while GSH, ATP, and taurine levels were negatively correlated with AST and ALT levels (Supplementary Fig. 4). At the same time, we found that the levels of some serum metabolites, namely VLDL/LDL, 2-hydroxybutyrate, citrate, and fumarate, were positively correlated with glutathione levels (data not shown). All results showed that ATP, taurine, glucose, and GSH in the liver, as well as serum lipids, 2-hydroxybutyrate, citrate, and fumarate were characteristic metabolites involved in chronic l-lactate exposure, suggesting that oxidative stress and energy metabolism alterations are involved in hepatic dysfunction.

**Fig. 4:**
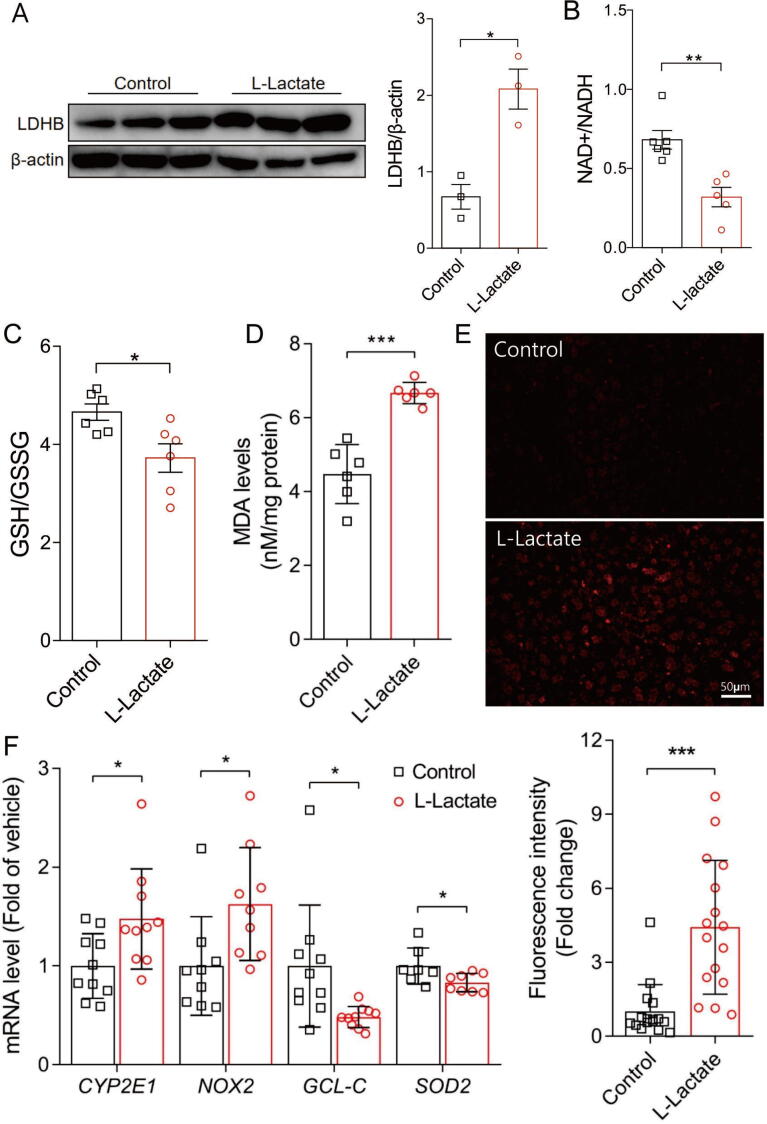
Chronic l-lactate exposure markedly increased hepatic lactate-pyruvate reaction and ROS accumulation due to impaired redox homeostasis. (A) Western blot of LDH-B protein and its quantitative analysis (n = 3). (B) NAD^+^/NADH levels (n = 6). (C) Ratio of hepatic glutathione (GSH) to oxidized glutathione disulfide (GSSG). (n = 6). (D) Malondialdehyde (MDA) levels (n = 6). (E) Representative images of dihydroethidium (DHE) staining in liver sections, and quantitative analysis of fluorescent intensity (n = 3). (F) Hepatic mRNA levels of ROS-generating genes (cytochrome P450 2E1 [*Cyp2e1*], NADPH oxidase-2 [*Nox2*]), and antioxidant genes (c-glutamylcysteine ligase catalytic subunit [*Gclc*], superoxide dismutase-2 [*Sod2*]) (n = 7–10). Data are expressed as mean ± S.D. *P < 0.05, ** *P* < 0.01, and *** *P* < 0.001.

### 3.6 Chronic l-lactate exposure markedly increases hepatic ROS levels owing to imbalanced redox homeostasis

After its transport inside the cell, following the reaction lactate is converted into pyruvate by the isoform B of the LDH: NAD^+^ + lactate = pyruvate + NADH, which directly changes the cytoplasmic NAD^+^/NADH ratio and redox state [6]. In this experiment, we observed that LDHB and NAD^+^/NADH in liver increased significantly after l-lactate exposure, suggesting the conversion of l-lactate to pyruvate (Fig. 4A-B). At the same time, we found that l-lactate exposure led to the decline of key enzymes of TCA cycle, i.e. citrate synthesis (CS), isocitrate dehydrogenase (IDH2), 2-ketoglutarate dehydrogenase (OGDH) (Supplementary Fig. 5), which was consistent with the changes of metabolite, i.e., ATP, succinate and fumarate, suggesting the inhibition of TCA cycle.

**Fig. 5:**
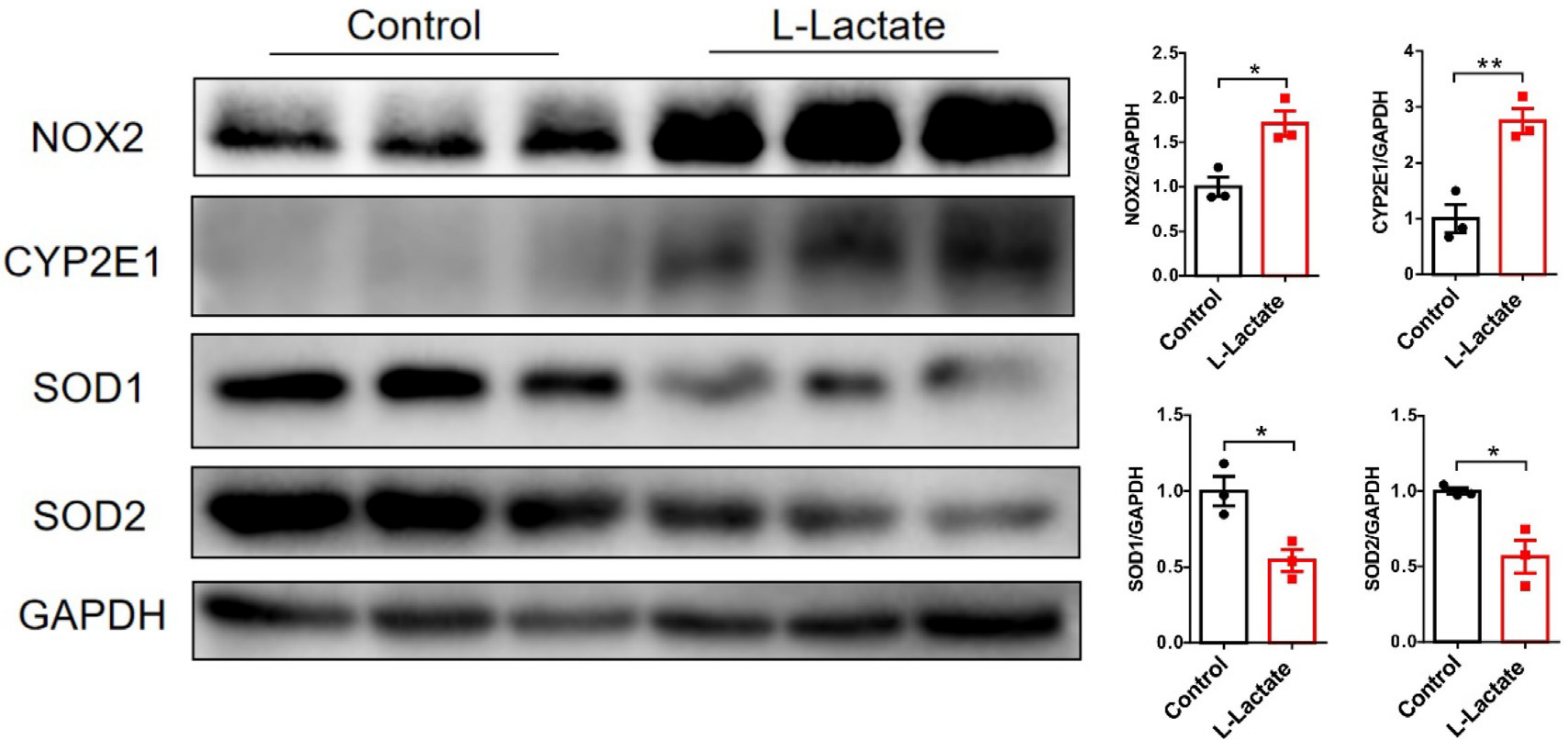
Western blots and quantitative analysis were performed for CYP2E1, Nox2, SOD1 and SOD2 in hepatic extracts of the mice (n = 3). Data are expressed as mean ± S.D. *P < 0.05, ** *P* < 0.01.

To further explore the potential mechanisms of liver damage induced by chronic l-lactate exposure, we examined the redox-sensitive indicators in mouse livers (Fig. 4C-F). Compared with the tissues from the control group, the tissues from the l-lactate group showed a significant reduction in GSH/GSSG levels (Fig. 4C); a significantly higher MDA level was also observed in the l-lactate group compared to the control group (Fig. 4D). Microscopy showed that hepatic sections from l-lactate mice exhibited a widespread and marked increase in DHE fluorescence compared with those from control mice, indicated ROS production (Fig. 4E). Furthermore, PCR was used to detect the ROS generating and scavenging systems (Fig. 4F). l-lactate exposure increased the expression of prooxidant genes (cytochrome P450 2E1 [Cyp2e1], NADPH oxidase-2 [Nox2]) and decreased the expression of antioxidant genes (c-glutamylcysteine ligase catalytic subunit [Gclc], superoxide dismutase-2 [Sod2]). At the same time, the protein level changes of the indicator are consistent with the mRNA changes (Fig. 5). Together, these data indicate the presence of increased ROS levels after chronic l-lactate exposure, resulting from redox alterations following l-lactate to pyruvate reaction.

### 3.7 Chronic l-lactate exposure induces ROS-mediated hepatic cell apoptosis via a mitochondrial pathway

**Fig. 6:**
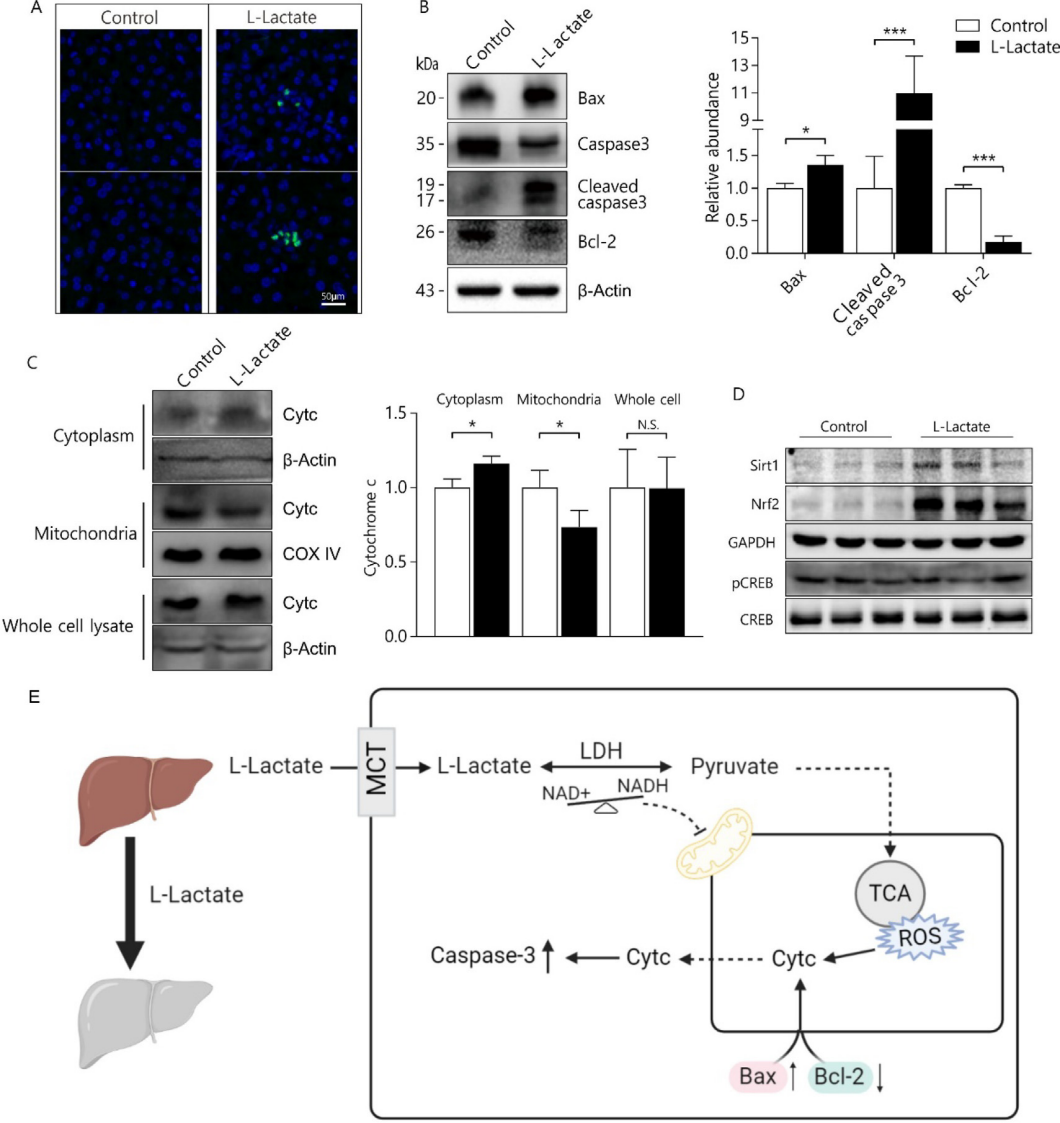
Chronic l-lactate exposure induces ROS-mediated hepatic cell apoptosis via a mitochondrial pathway. (A) TUNEL staining of livers (n = 3). (B) Western blots were performed for Bax, caspase 3, cleaved caspase 3 and Bcl-2 in liver extracts (n = 3). (C) Determination of cytochrome *c* (Cytc) levels in cytoplasm, mitochondria, and whole-cell hepatic extracts (n = 3). (D) Western blots were performed for Sirt1, Nrf2, and p-CREB in hepatic extracts (n = 3). (E) Schematic diagram of lactate-pyruvate reaction and mitochondrial related cell apoptosis involved in lactate exposure. Data are expressed as mean ± S.D. *P < 0.05 and *** *P* < 0.001 compared to control.

To further study the potential molecular mechanisms of l-lactate in hepatic tissues, we next examined the expression patterns of Cytc, Bax, Bcl-2, and cleaved caspase-3. First, TUNEL-FITC staining showed that the number of apoptotic nuclei was markedly increased in the l-lactate mice (Fig. 6A). In addition, compared to those of controls, livers of the l-lactate mice had significantly increased protein expression of Bax and decreased expression of the anti-apoptotic protein Bcl-2 (Fig. 6B). After l-lactate exposure, the expression of hepatic Cytc increased in the cytoplasm and decreased in the mitochondria, while there was no significant change in Cytc expression in the total cell lysate (Fig. 6C). The release of Cytc from the mitochondria into the cytoplasm, implies that caspase-3 is activated. Accordingly, l-lactate markedly increased hepatic cleaved caspase-3 expression in a Cytc-mediated manner. In addition, we detected increased protein levels of Sirt1 in the l-lactate mice, whereas p-CREB levels were not changed, suggesting that the GPR81-cAMP-CREB pathway is not involved in liver injury induced by l-lactate exposure (Fig. 6D).

## 4 Discussion

In this study, the effects of l-lactate administration on liver, kidney, muscle, and serum samples and the mechanism underlying these observed effects were investigated in a mouse model of chronic l-lactate exposure. During chronic l-lactate exposure, accumulation of l-lactate takes place when the rate of exogenous l-lactate supplementation exceeds the cellular capacity for the removal of l-lactate by pyruvate dehydrogenase and mitochondrial oxidation. l-lactate is cleared and transformed to glucose, primarily by the liver [36], as well as skeletal muscle [37], kidney [38], brain [1]. The relationship between plasma lactate and glucose concentrations is defined as Cori cycle by Cori and his colleagues [39], [40]. This cycle of substrate fluxes, simplified as plasma glucose/muscle glycogen/plasma lactate/liver glycogen/plasma glucose, and the changed blood glucose levels we observed (Fig. 1B), suggested the two states of gluconeogenesis after l-lactate treatment.

The specific effects of l-lactate on peripheral organs must be investigated separately. Both histopathological and serum biochemical analyses conducted in this study showed that l-lactate manifestly impaired hepatic function and morphology but did not affect kidney and muscle tissues; PAS staining also revealed glycogen deposition in liver tissues. All experiments suggested that l-lactate accumulation may be associated with morphological and functional changes in the liver, but not other tissues. Our results confirmed the hypothesis that liver injury is dependent, at least in part, on l-lactate accumulation [16].

On visually inspecting metabolic profiles and considering the values of R^2^Y and Q^2^Y in OPLS-DA, we observed distinct clusters of metabolites mainly in liver and serum samples from mice undergoing vehicle and l-lactate treatment. By integrating results from the loading plot (Fig. 3B) and statistical analysis of metabolites (Supplementary Table 3), we determined that chronic l-lactate exposure contributes to changes in the levels of bioenergy and redox metabolites such as taurine, GSH, glucose, pyruvate, and adenosine metabolites (ATP/ADP/AMP) in liver tissues, as well as lipids (VLDL/LDL, phosphocholine, and 2-hydroxybutyrate) in serum. When lipid metabolism disorder occurs, lipids accumulate in the liver and are released into the blood [41]. Therefore, changes in serum lipids also indicate hepatic dysfunction. In addition, the increase in hepatic glucose content suggests an enhancement of glycogenesis. Additional correlation analysis indicated that taurine and glutathione were most closely related to liver injury after chronic l-lactate exposure.

GSH, which is biosynthesized from cysteine, glycine, and glutamate, is the most abundant cellular redox molecule and plays an important role in antioxidation during long-term physical exercise [42], [43]. In addition, taurine is an effective antioxidant that is implicated in oxidative damage, endoplasmic reticulum stress, and cell apoptosis [44], [45]. We found that the levels of both GSH/GSSG and taurine were significantly reduced in the livers after l-lactate accumulation, indicated alterations in redox state. Among the diverse consequences of increasing intracellular l-lactate levels in an MCT-dependent manner, the redox state also changes according to l-lactate conversion into pyruvate under the activity of LDH-B [46]. When l-lactate is converted to pyruvate there is a change in redox state as l-lactate is more reduced than pyruvate [6]. In fact, the intracellular l-lactate shuttle mechanism is vital for distributing energy substrates and redox balance from the cytosol to the mitochondria [8], [47], [48].

Redox homeostasis in response to environmental stimuli is essential for normal cellular physiology, and perturbation in this homeostasis contributes to changes in redox metabolism and pathology [49]. Under normal conditions, the formation of ROS and its removal by endogenous antioxidant molecules are in a homeostatic balance [50]. In this study, l-lactate exposure was found to lead to significantly increased hepatic levels of MDA and ROS and decreased GSH/GSSG and taurine levels. Furthermore, our results revealed decreased mRNA levels of Sod2 and GLS-2, which are antioxidant markers [45], and increased levels of Cyp2e1 and Nox2, which are markers of lipid pro-oxidation [35], [51], in the hepatic tissues of the l-lactate mice. Therefore, our data indicated that hepatic oxidative stress might be the result of ROS overproduction under impaired antioxidant capacity after chronic l-lactate exposure.

ROS are mainly produced in the mitochondria of cells and can influence the mitochondrial membrane potential, trigger the release of Cytc, and induce caspase 3-dependent apoptosis [52]. Generally, the Cytc-mediated mitochondrial apoptosis pathway is also controlled by the anti-apoptotic Bcl-2 and pro-apoptotic Bax [53]. In this study, we detected changes in fluorescence intensities of hepatocytes and observed that l-lactate exposure enhanced the rate of apoptosis. Our study further showed that chronic l-lactate exposure stimulates Bax/Bcl-2 activation, triggers Cytc release, and promotes caspase-3 cleavage and apoptosis in hepatocytes, ultimately leading to the occurrence of hepatic pathologies (Fig. 6D).

The shift in cellular redox state can be characterized by the NAD^+^/NADH ratio, which has been shown to increase NAD^+^-dependent Sirt1 expressions [16]. Besides, it was reported that lactate mediates the antilipolytic action of insulin through activation of the Gi-coupled receptor GPR81 and decrease in cAMP levels [54]. However, we did not find significantly decreased levels of p-CREB in l-lactate group, suggested that the GPR81-dependent p-CREB signal was not involved in the effects of l-lactate on hepatocytes.

This study provides *in vivo* evidence that lactate exhibits redox-mediated pro-apoptotic effects in the hepatocyte. To our knowledge, this is the first study addressing the pharmacological role of an substrate of carbohydrate metabolites in the liver and therefore opens new field of investigation that related to hyperlactatemia, such a condition is observed during physiological exercise, as well as in pathological conditions (e.g., hypoxia, diabetes, and cancer).

Several limitations should be considered: firstly, although mice and human beings share similarities in lactate production and usage pathways, differences exist particularly as they apply to mechanistic aspects of physiology and pathology involved in hyperlactatemia. Secondly, the alterations of blood glucose levels after l-lactate exposure may be related to the decrease of gluconeogenic ability of hepatocytes, the changed Cori cycle needs further studies.

## 5 Conclusions

In summary, in this study, histopathological and serum biochemical analyses showed that chronic l-lactate exposure can significantly affect hepatic function in mice. Metabolomic analysis indicated that the redox metabolites taurine and glutathione were involved in hepatic dysfunction. Furthermore, molecular biology analysis revealed that the mechanism underlying this hepatic dysfunction was mainly the imbalance of NAD^+^/NADH dependent redox homeostasis, as well as the mitochondria-mediated apoptotic pathway via upregulation of the Bax/Bcl-2 ratio, release of Cytc and activation of caspase-3.

## Author contributions

LZ. and HG. contributed to experimental design and writing of the manuscript. MD. and QY. contributed to animal experiment data acquisition. DS., HJ., JX., JY., LZ. contributed to data analysis and result interpretation. All authors have read and approved the final manuscript.

## Declaration of Competing Interest

The authors declare that they have no known competing financial interests or personal relationships that could have appeared to influence the work reported in this paper.
