# Supplementary material for: A combined metabolomics and molecular biology approach to reveal hepatic injury and underlying mechanisms after chronic l-lactate exposure in mice
Source: Comput Struct Biotechnol J. 2022 Jul 25;20:3935–45. doi: 10.1016/j.csbj.2022.07.034 (PMC9352416; doi:10.1016/j.csbj.2022.07.034)
Supplement: Supplementary data 1 [file mmc1.docx]

**Supplemental Information**

**Supplemental table 1**. Sequences of PCR primers.

| Gene | Gene Bank  Accession Number | Primer Sequence |
| --- | --- | --- |
| *CYP2E1* | NM_021282 | F 5'-TGTGACTTTGGCCGACCTGTTC-3'  R 5'-CAACACACACGCGCTTTCCTGC-3' |
| *Nox2* | NM_007807 | F 5'-GAAAACTCCTTGGGTCAGCACT-3'  R 5'-ATTTCGACACACTGGCAGCA-3' |
| *Gpx1* | NM_008160 | F 5'-CCAGGAGAATGGCAAGAATGA-3'  R 5'-TCTCACCATTCACTTCGCACTT-3' |
| *G6pdx* | NM_008062.3 | F 5'-GAGTGGGCTTCCAGTACGAG-3'  R 5'-GCCTATGGTCCCAAAGTCCT-3' |
| *GAPDH* | NM_001289726.1 | F 5'-GGCATCGTGGAAGGGCTCATGAC-3'  R 5'-ATGCCAGTGAGCTTCCCGTTCAGC-3' |

**Supplemental table 2**. Summary of the parameters of PCA and OPLS-DA models from different groups of the mice.

|  | PCA models | |  | OPLS-DA models | | | |  |
| --- | --- | --- | --- | --- | --- | --- | --- | --- |
| Groups | *No*^a^ | *R*^2^X(cum)^b^ |  | *No*^a^ | *R*^2^X(cum)^b^ | *R*^2^Y(cum)^b^ | *R*^2^Q(cum)^b^ | *p value* |
| Serum | 2 | 0.550 |  | 1P + 1O | 0.543 | 0.952 | 0.865 | 0.001 |
| Muscle | 2 | 0.517 |  | 1P + 1O | 0.411 | 0.875 | 0.098 | 0.907 |
| Kidney | 2 | 0.455 |  | 1P + 1O | 0.324 | 0.949 | 0.596 | 0.062 |
| Liver | 2 | 0.601 |  | 1P + 1O | 0.583 | 0.911 | 0.795 | 0.004 |

^a^Number of components. 1P + 1O, one predictive component and one orthogonal component for constructing the OPLS-DA model.

^b^*R*^2^X(cum) and *R*^2^Y(cum) are the total explained variance in X and Y matrix, respectively, and *R*^2^Q(cum) is the cross-validated predictive ability in Y matrix. The values of these parameters close to 1.0 indicate a robust mathematical model with a reliable predictive accuracy.

**Supplemental table 3.** Fold change of metabolites in different tissues from L-lactate mice compared to the controls.

| No. | Metalolites | Serum | | Muscle | | Kidney | | Liver | |
| --- | --- | --- | --- | --- | --- | --- | --- | --- | --- |
|  |  | Fold  change | \|r\| | Fold  change | \|r\| | Fold  change | \|r\| | Fold  change | \|r\| |
| 1 | VLDL/LDL | -24.63^**^ | 0.67 |  |  |  |  |  |  |
| 2 | Leucine | 3.27 |  | 0.04 |  | -3.15 |  | 5.04 |  |
| 3 | Isoleucine | -0.15 |  | 2.12 |  | -3.32 |  | 2.19 |  |
| 4 | Valine | -3.05 |  | 3.44 |  | -5.53 |  | 14.52 |  |
| 5 | Isobutyrate | -16.92 |  |  |  |  |  |  |  |
| 6 | 2-hydroxybutyrate | -45.68^**^ | 0.74 | -16.89 |  | -26.09^*^ | 0.61 | -34.20^*^ | 0.67 |
| 8 | Alanine | 19.21 |  | -1.72 |  | 4.94 |  | -0.69 |  |
| 9 | Acetate | -7.82 |  | 39.32 |  | 2.42 |  | -20.96^**^ | 0.75 |
| 10 | Lipid | -13.54^*^ | 0.73 |  |  |  |  |  |  |
| 11 | Glutamate | -34.73 |  | -28.64^*^ | 0.62 | 12.03^***^ | 0.80 | 4.44 |  |
| 12 | Pyruvate | 30.93^*^ | 0.56 | -0.56 |  |  |  |  |  |
| 13 | Succinate | -7.35 |  | 44.06^*^ | 0.56 | -5.28 |  | -28.77^*^ | 0.63 |
| 14 | Glutamine | 2.72 |  | -9.43 |  | 8.36 |  | -15.87 |  |
| 15 | Citrate | -23.50^*^ | 0.66 |  |  |  |  |  |  |
| 16 | Glutathione |  |  | -2.52 |  |  |  | -30.20^***^ | 0.82 |
| 17 | Aspartate |  |  |  |  | 0.47 |  |  |  |
| 18 | Creatine | 2.38 |  | -0.87 |  | 3.29 |  | -6.38 |  |
| 19 | Choline | 13.36 |  | 13.23 |  | -2.45 |  | -17.11^*^ | 0.68 |
| 20 | Phosphocholine | 41.16^*^ | 0.59 | -17.92 |  | 12.54^***^ | 0.82 | -13.01^*^ | 0.76 |
| 21 | GPC | 4.29 |  | 8.70 |  | 11.89 |  | -12.16^***^ | 0.86 |
| 22 | Taurine |  |  | -0.85 |  | -11.62^***^ | 0.90 | -29.80^**^ | 0.74 |
| 23 | Glucose | -6.87 |  | 7.11 |  | -10.70^*^ | 0.56 | 7.36^**^ | 0.77 |
| 24 | Glycine | 10.83 |  | 13.83^*^ | 0.69 | 6.39^*^ | 0.62 | -12.27^**^ | 0.72 |
| 25 | Urea | -14.43 |  |  |  |  |  |  |  |
| 26 | Myo-inositol |  |  |  |  | 12.12^*^ | 0.61 |  |  |
| 27 | Phosphocreatine |  |  | 19.81^*^ | 0.74 |  |  |  |  |
| 28 | Fumarate | -39.32^*^ | 0.56 | -43.74^**^ | 0.71 | -7.98 |  | -30.21^**^ | 0.60 |
| 29 | Tyrosine | 47.84^*^ | 0.62 |  |  | 5.98 |  | 2.22 |  |
| 30 | Histidine | 15.17 |  | 14.46 |  |  |  | -14.42 |  |
| 31 | Phenylalanine | 7.09 |  | -6.01 |  |  |  | 9.49 |  |
| 32 | Uridine |  |  |  |  | -6.67 |  | -4.59 |  |
| 33 | ADP |  |  |  |  | 5.95 |  | -38.42^***^ | 0.82 |
| 34 | ATP |  |  |  |  | -0.81 |  | -44.26^**^ | 0.78 |
| 35 | IMP |  |  | -8.70 |  | 27.38 |  | -44.34^***^ | 0.88 |
| 36 | AMP |  |  | -4.21 |  | 43.00 |  | -49.04^***^ | 0.87 |
| 37 | Histimine |  |  | 11.53 |  |  |  |  |  |
| 38 | Niacinamide |  |  | -30.39^*^ | 0.69 | -6.89 |  | -32.36^***^ | 0.88 |

^a^ Keys: **P* < 0.05, ***P* < 0.01, ****P* < 0.001, independent samples Student’s t-test; the loading (p) correlation coefficients were calculated from OPLS-DA results, illustrated by |r| value.


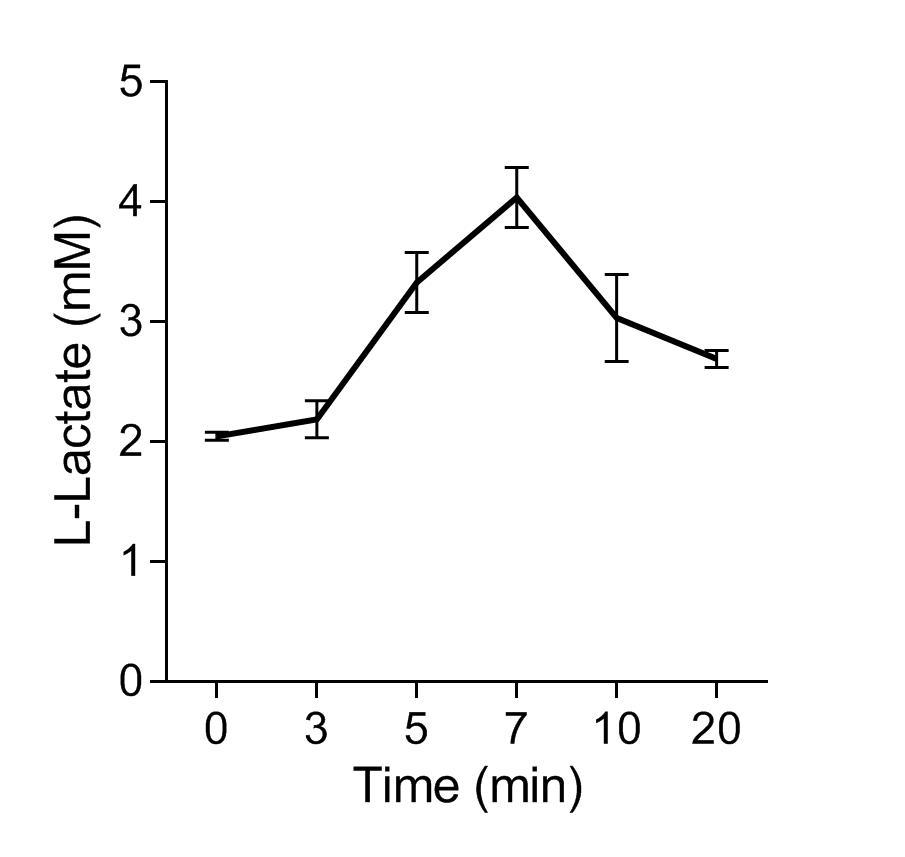


**Supplemental figure 1.** Acute L-lactate administration (1 g/kg, i.p.) increases blood lactate concentration (n = 7). Data are the mean ± S.D.


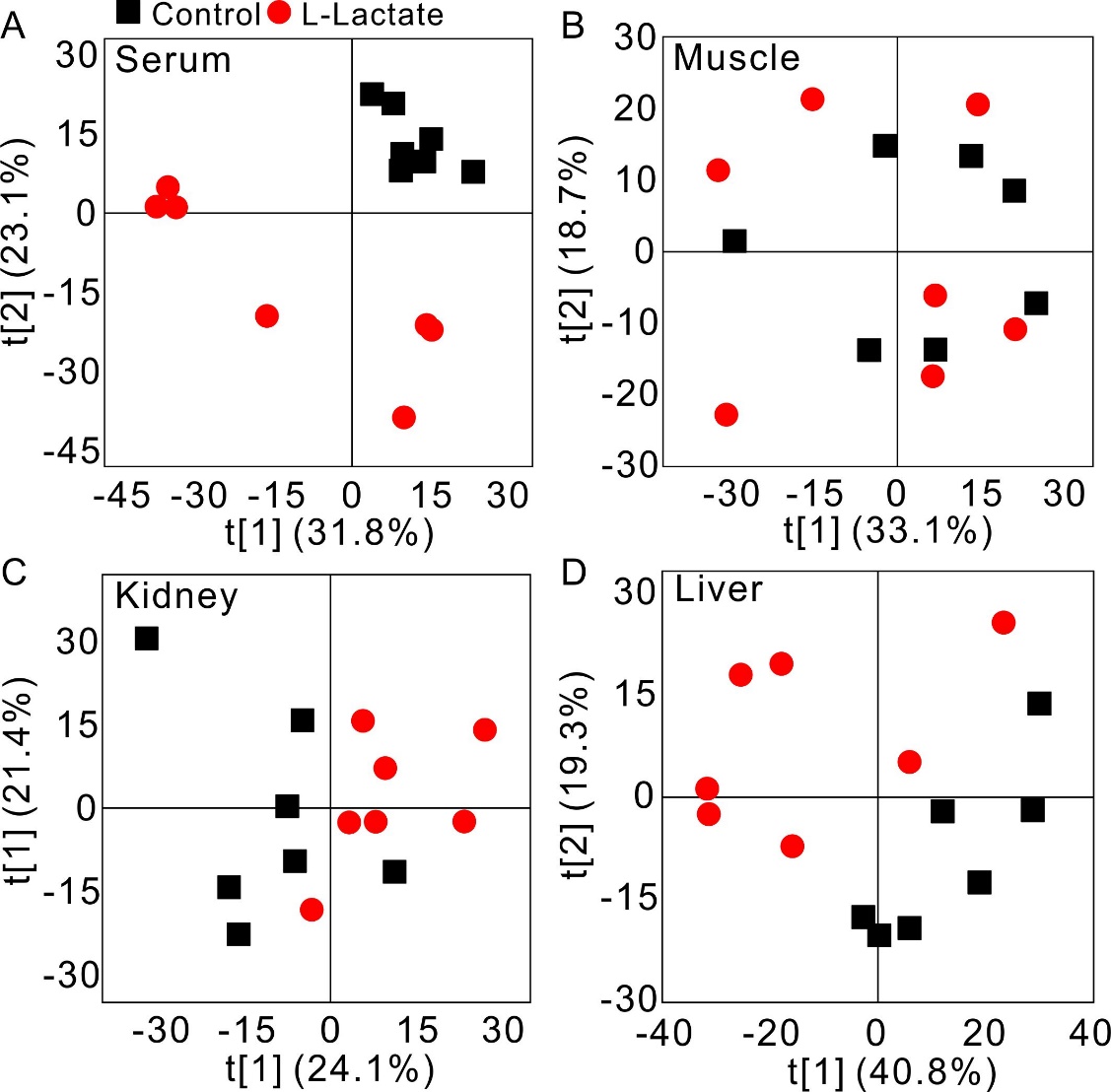


**Supplemental figure 2.** Score plot of principal component analysis (PCA) in serum (A), muscle (B), kidney (C), and liver (D) between control and L-lactate treated mice.


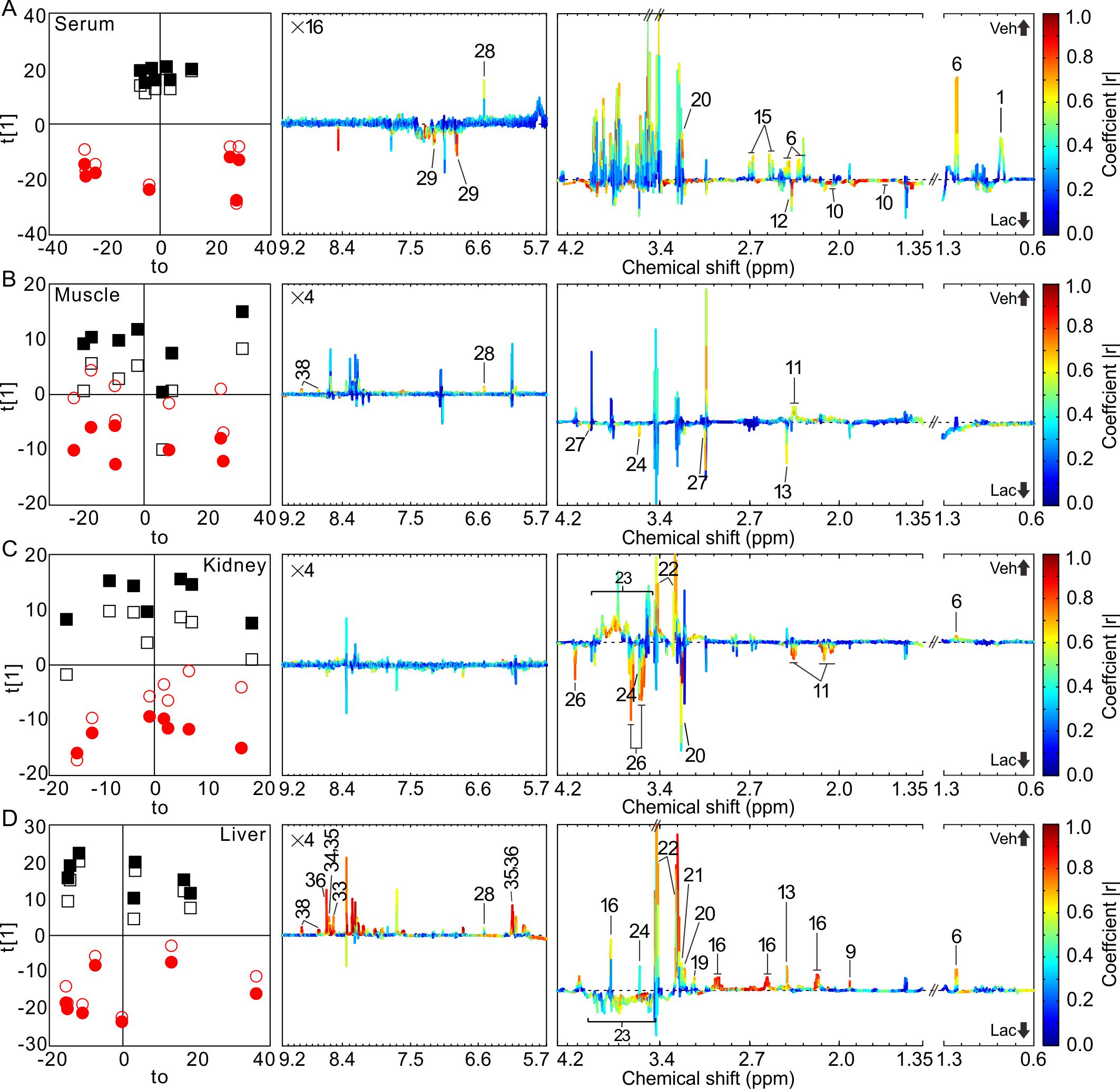


**Supplemental figure 3.** Cross-validated OPLS-DA score plot (left) and coefficient-coded loading plots (right) for the models discriminating the control (blank squares) and lactate-treated groups (red dots) for data obtained from serum (A), aqueous extracts of muscle (B), kidney (C), and liver (D). Cross-validated score value expressed with a hollow symbol. Metabolites assignment refers to Supplemental Table 3.


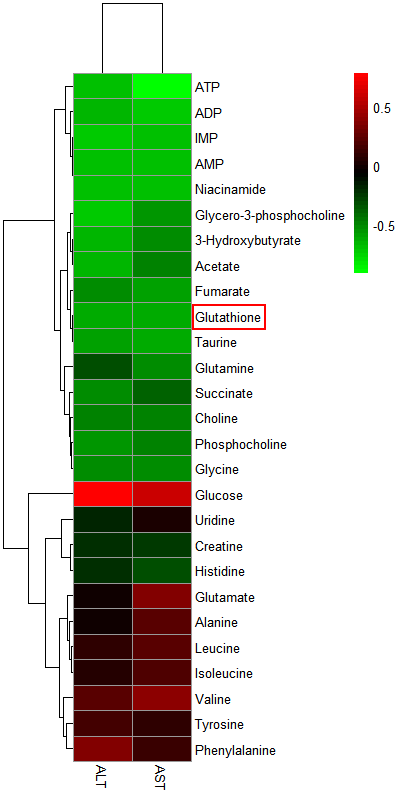


**Supplemental figure 4.** Correlation analysis between hepatic metabolites and serum alanine transaminase/aspartate transaminase (ALT/AST) levels. The glutathione, ADP, AMP, ATP was negatively correlated, while glucose was positively correlated with ALT/AST indicators, respectively.


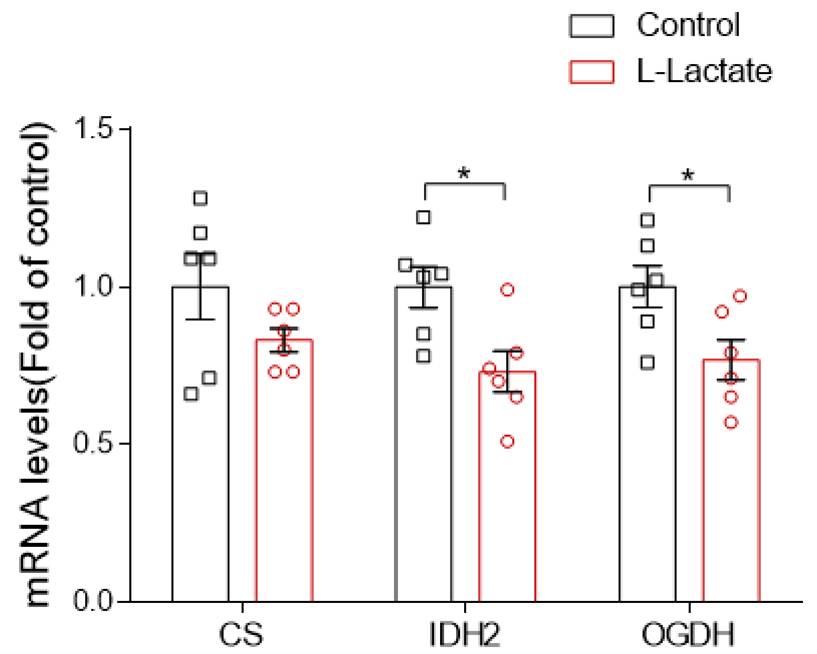


**Supplemental figure 5.** RT-PCR determination of TCA cycle enzymes in hepatic extracts from l-lactate and control mice. Data are expressed as mean ± S.D. n = 6. **P* < 0.05.
